# Optimizing process and methods for a living systematic review: 30 search updates and three review updates later

**DOI:** 10.1016/j.jclinepi.2023.111231

**Published:** 2024-02

**Authors:** Ailsa R. Butler, Jamie Hartmann-Boyce, Jonathan Livingstone-Banks, Tari Turner, Nicola Lindson

**Affiliations:** a Nuffield Department of Primary Care Health Sciences, University of Oxford, Oxford, UK; b Cochrane Australia, School of Public Health & Preventive Medicine, Monash University, Melbourne, Australia; c Department of Health Promotion and Policy, University of Massachusetts, Amherst, MA, USA

**Keywords:** Evidence synthesis, Systematic review, Living systematic review, Living evidence, Methods for planning and reporting, Methods for living systematic review, Living guidelines

## Abstract

**Objective:**

To describe the living systematic review (LSR) process and to share experience of planning, searches, screening, extraction, publishing and dissemination to inform and assist authors planning their own LSR. Many LSR do not publish more than one update, we hope this paper helps to increase this.

**Study Design and Setting:**

A Cochrane LSR with an international author team that has been ‘living’ for two years, with monthly search updates and three full updates published in this time. LSRs are regularly updated systematic reviews that allow new evidence to be incorporated as it becomes available. LSR are ideally suited to policy-relevant topics where there is uncertainty and new evidence will likely impact the interpretation and/or certainty of outcomes.

**Results:**

The key features of the process that require consideration are: specifying the frequency of searches and triggers for full updates in the protocol; stakeholder input; publishing and disseminating monthly search findings. A strong team, incorporating methodological and topic expertise, with core members that meet regularly is essential. Regular search updates make it important to have a clear cyclical schedule of activity. To achieve timely updates this process should be streamlined, for example, using automated monthly searches, and systematic reviewing software for screening. LSR provide a unique opportunity to incorporate stakeholder feedback.

**Conclusions:**

We recommend that LSRs should be: justified; carefully planned including the timing of search updates, triggers for publication and termination; published in a timely manner; have a clear dissemination plan; and a strong core team of authors.

**What is new?**

**Key findings**
• Living systematic reviews (LSR) suit fast moving policy relevant healthcare topics. More than half of published LSR do not publish an update we aim to increase this [1].

**What this adds to what was known?**
• We share LSR methodology & practical steps to make the process transparent.
• We share strategies for dissemination of living review findings.

**What is the implication and what should change now?**
• Increase the proportion of LSR publishing more than once.
• Timely updates allow stakeholders access to recent evidence for decision-making.

## 1 Introduction

The living systematic review (LSR) process was first proposed in 2014. It provides a way to regularly update systematic reviews as new evidence becomes available, using the same established, rigorous, and recommended methods used in standard systematic reviews [1]. LSRs allow for timely dissemination of research findings and link evidence to practice. They are relevant to priority areas where there is uncertainty, and research is rapidly emerging. These factors mean that changes in review findings or the certainty of the evidence are likely and will impact on the decision-making of stakeholders. For these reasons, the COVID-19 pandemic resulted in a proliferation of LSR [2].

LSR methods are well suited to the relatively recent, fast-moving nature of e-cigarette (EC) research. Combustible cigarettes are the leading preventable cause of illness and death worldwide [3]. Uncertainties surrounding the effects of EC for quitting smoking hampers policy, clinical, and personal decisions. Thus, we developed an LSR from a standard Cochrane Review of EC for smoking cessation. The review was first published in 2014 and became an LSR in 2020. From first publication to the present day, the review has informed policy worldwide.

Although there was much public and policy interest in the topic pretransition to LSR, there were few relevant randomized controlled trials and these randomized controlled trials tested EC with outdated technology. This resulted in evidence with imprecision and indirectness, and Grading of Recommendations Assessment, Development and Evaluation ratings of low and very low certainty for most outcomes [4,5]. Transitioning to the LSR approach allowed us to capture new evidence as it was published, feed it into our findings and our certainty in these, and allow stakeholders access to the most accurate, up-to-date information.

However, LSRs are labor intensive and it is important to know when implementing their methods is justified. Evidence suggests that more than half of published LSRs have not completed at least one update [2,6]. Often standardized registration is not completed, plans change during the process, research is not reported in-line with protocols, and the frequency or triggers for updates are not specified [2,6].

There are useful guidance and methods papers available on the LSR approach [[7], [8], [9], [10], [11], [12], [13], [14], [15], [16], [17], [18]]. This paper plays a complementary role by outlining practical steps specific to our process, reflecting on lessons we have learnt along the way. Through carrying out our LSR, we have noted the crucial importance of following a set of prespecified steps in a timely fashion. A member of our author team who specializes in the LSR approach (T.T.) encouraged us to share our methods to assist other authors, with the aim of increasing the proportion of LSRs publishing updates to their reviews. By sharing our experience, we also hope to both demystify and provide an overview of the LSR process to assist researchers who may be considering undertaking LSR to decide whether this is a feasible and appropriate approach for them.

## 2 Study design and setting

A Cochrane LSR with an international author team that has been ‘living’ for two years. LSRs are regularly updated systematic reviews that allow new evidence to be incorporated as it becomes available. To inform and assist authors planning their own LSR by describing our process and experiences when carrying out an LSR, covering planning, searches, screening, extraction, publishing, and dissemination.

## 3 Results

**Fig. 1:**
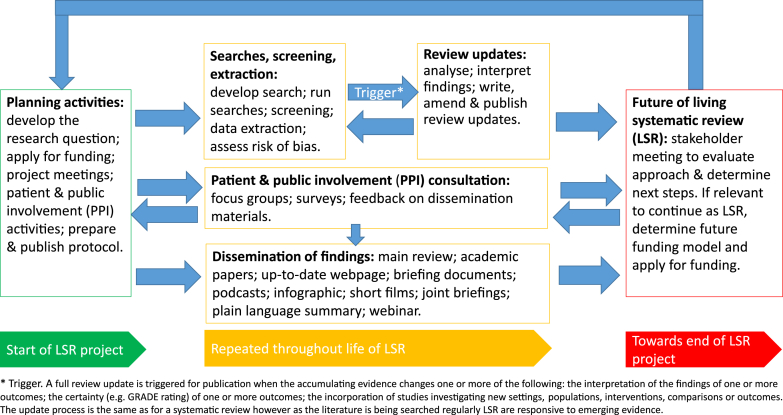
Flow diagram of activities to summarize the living review (LSR) process. (For interpretation of the references to color in this figure legend, the reader is referred to the Web version of this article.)

We describe the steps in our LSR process from seeking and incorporating stakeholder input at the outset and during the process, developing key aspects of our protocol and search strategy, managing monthly searches, publishing review updates, disseminating our findings, and reviewing the future status of the LSR (Fig. 1).

### 3.1 Planning activities

#### 3.1.1 Stakeholder and public involvement

At the outset of any systematic review, it is important to map out the main audiences. Our stakeholders include policy makers, health professionals, guideline developers, governments, researchers, research funders, and members of the public. In 2016, we undertook a stakeholder engagement project related to the wider topic of tobacco control; EC emerged as the highest priority topic [19]. Potential LSR authors could draw on similar exercises to inform their topics and outcomes [20].

We consulted with members of the public throughout our LSR process, as prespecified in our funding application (our LSR is funded by Cancer Research UK) and LSR protocol. We carried out two online surveys advertised via social media. The first, LSR launch (2020), asked for feedback on the review scope and optimum methods for sharing information. We received 394 responses. The second, in 2022, evaluated the usefulness and future of the LSR and associated dissemination materials. We had 197 responses; Supplementary Table 1 includes the survey questions and summarizes responses. We also held focus group meetings with members of the public in 2020, 2021, and 2022, with clear discussion points on the LSR scope, dissemination methods, and future of the LSR. Views of focus group members were anonymously noted and summarized. Our core group of public contributors, who smoke or have smoked in the past, comment on our dissemination outputs regularly.

The LSR method allows for new outcomes of relevance to be incorporated in updates of the review if these are prespecified, reported with transparency and are free from bias. Collaboration with stakeholders allows us to identify and monitor new outcomes of relevance. For example, working with policy partners and drawing on feedback from the focus groups mentioned above led us to identify longer-term use of EC and other quitting aids as a new outcome. We prepublished our intention to add this outcome on the Cochrane Library and incorporated it into the LSR in the following update.

#### 3.1.2 Protocol

Our LSR was based on an existing systematic review that had a protocol, published before review screening began. Before LSR project launch, we added an appendix to the latest update of our review to specify the methods particular to the LSR process. This was published before the new LSR process started [21]. Whether a review is starting out as an LSR or is transitioning into an LSR, methodology should be in the public domain before the process begins.

We incorporated protocol elements, specific to the conduct of an LSR shown in Table 1.

**Table 1:** Protocol elements specific to LSR; considerations for triggering an update; actions following monthly searches and tips for conducting an LSR

1. Protocol elements specific LSRs	Examples from our LSR	Alternative options
Justification for the ‘Living Review’ status	Uncertainty around EC use as a quitting tool and safety when used for this purpose.	Specific to other topics and research questions
When LSR methodology will become active, and whether there is a predefined period that the review will remain ‘living’	We specified when our EC review would become living, which was published as an appendix to the Cochrane Review in the Cochrane Library prior to it becoming ‘living’. We also specified the minimum time that the review would be ‘living’ and when we would assess its future.	Publish a protocol containing this information on an open access repository or in a journal
How often search updates will be carried out	Our searches are carried out monthly	This could be weekly three monthly, 6 monthly or at any other interval; dependent on how often new evidence is likely to become available in the relevant topic
Any process in place for reviewing the search strategy during the LSR project	The search terms will be updated as necessary to include terms referring to EC or vapes as the terminology evolves	Assess the changing landscape of the particular review topic to incorporate new terms when relevant.
Any specific methods that will be used for screening, data extraction, and synthesis, aimed at streamlining the process	Software for screening (Covidence); prepiloted data extraction sheets.	Alternative systematic reviewing software or alterations to the screening/extraction process used (e.g., only single screening of a percentage of references)
How/where the results of the regular searches will be shared	Monthly updates to stakeholder briefing documents hosted on project webpage; spreadsheet also linked to our webpage; monthly podcast; social media posts	Other rapid dissemination methods, for example, mailing lists, webinars
Effects of triggering a full update of the review to incorporate new evidence	We aim to update the review and analysis and submit for peer review and publish within 3 mo.	Set an appropriate process for and timeline within which to complete and publish the new update appropriate to the urgency of topic updates
Plans to monitor the applicability of the ‘living’ status of the review	If evidence is judged to be high certainty for all outcomes and all relevant comparisons, consideration will be given to ceasing LSR status. Where the originally planned project duration is approaching and outcomes are not yet certain, discussions will be held with the author team and stakeholders to determine next steps	At protocol stage decide what certainty of evidence is required, for which comparisons and outcomes, to no longer warrant regular updates of the literature. If research in the area begins to be published less regularly, it may also be appropriate to review the applicability of the format.

2. Considerations for triggering an update	
Trigger	Things to consider
(Possible) change to the interpretation of the findings of one or more outcomes	This can be challenging to determine without updating analyses. If the new study has an effect direction differing from that in the current analysis, the study could be entered into a meta-analysis to test the impact of its inclusion on the results. If the study has an effect congruent with that of the current analysis, but confidence intervals for the current analysis are wide, again the study could be entered into a meta-analysis to test the impact of its inclusion on the results. If there are no relevant meta-analyses, the study would contribute to, but other similar studies which are synthesized without meta-analysis, an update should be considered if the results of the new study are not congruent with the data synthesized to date.
(Possible) change to the certainty of one or more outcomes	This can also be challenging to determine without updating analyses, and will depend on the current GRADE ratings and the reasons for any downgrading, where relevant. For example, a new study might increase certainty if: • The current certainty is downgraded based on indirectness, and the new study is more directly relevant (e.g., a more recent EC device) • The current certainty is downgraded based on risk of bias, and a new study is judged to be at low risk of bias across all domains AND is congruent with the existing analysis • The current certainty is downgraded based on imprecision and the new study or studies include an informative number of participants for the outcome of interest, which is congruent with existing findings. A new study might decrease certainty if, for example: • It introduces unexplained statistical heterogeneity where previously there was none • Its findings are not congruent with results and it widens confidence intervals to the extent that findings are less certain because of imprecision
The incorporation of studies investigating new settings, populations, interventions, comparisons, or outcomes	It is worth reflecting from the start which new features of a study are relevant to decision makers, and hence might justify an update on this basis. This will be topic-dependent. For example, in our review, important new study features which led to consideration of an update were: • The first study to be conducted in pregnancy (new population) • Studies of newer e-cigarette devices (new interventions) • Studies comparing e-cigarettes based on flavor, nicotine content, and/or device type (new comparisons)

3. Monthly searches: outcomes and actions			
Possible outcomes of monthly searches ↓	Share numbers of eligible records identified (new study, report linked to already included study, or new ongoing study)	Extract data, assess quality	Trigger update
1) No new studies or information identified	Yes	No	No
2) New studies or information identified that has no important impact on review conclusions	Yes	Yes	No
3) New records identified that have an important impact on review conclusions	Yes	Yes	Yes

4. Tips for conducting an LSR	Examples from our LSR
• Stakeholder involvement to maintain relevance. Stakeholders may include members of the public, policy makers, healthcare professionals, and researchers	We have a PPI advisor, hold regular focus groups meetings, and surveys to gather views
• Strong core team that meets regularly	The core team of three researchers meet weekly to review time-sensitive tasks required to keep the living process on track
• Regular review of search strategy with input from an information specialist (IS)	The IS for this review is a topic expert who reviews the search strategy regularly to ensure that new terms, for example, for vapes, pods, etc. The researchers screening studies are also mindful of new terms they may encounter and feed this back to the IS
• Automated monthly searches	Our monthly automated searches mean that searches are always carried out on the first of the month regardless of holidays or weekends and save on researcher time
• Use of systematic reviewing software to aid screening	We use Covidence which is free to access for Cochrane Reviews (https://www.covidence.org/)
• Set deadlines for screening search results	Our search results are screened within 1 wk of each monthly search
• Keep search findings batched to allow a review update to be edited and published, although searches are being carried out for subsequent updates	Our searches are carried out monthly and each month stored as a separate review on Covidence
• Develop and communicate clear rules to trigger an update	We clearly specified the conditions under which a new update would be triggered in an appendix to the review before the living review process began. See Table 1 section 2 above
• Develop and communicate ‘stopping rules’ to interrupt the update process if studies are found that will change key conclusions between triggering the update and publication	We developed the following stopping rules. That would halt the production of an update. These were published in the appendix of the review before the LSR process commenced. Evidence is identified that (1) will change the certainty of our smoking cessation outcome, (2) will change the direction of effect for our smoking cessation outcome, (3) will provide new signal of a serious adverse event (SAE) attributable to EC, and (4) will change the direction of effect for the SAE outcome so it indicates harm or leads to certainty being downgraded where the effect direction had indicated benefit for comparisons: EC vs. NRT; non-nicotine EC vs. nicotine EC; EC vs. behavioral or no support
• Consider making justified and transparent methodological changes, for example, adding new outcomes, in response to stakeholder input	We prepublish any changes before these are included in an update to the review. For example, longer term use of EC or other study product at 6 months or longer was added as a new outcome in response to stakeholder feedback. With enough notice, the publication of planned change is included in the most recent published version of the review. If the change is decided upon after the publication of the latest version and needs to be included in the next version, then a protocol is published on an open-access online repository.
• Have a clear dissemination strategy. Consider regularly updated, freely available, stakeholder briefing documents with an information box to easily update search findings.	See Table 2
• Assess future need to maintain review as an LSR	The full author team review the LSR status at our full team meetings which occur every 6–9 mo.

*Abbreviations*: LSR, living systematic review; EC, e-cigarette; GRADE, Grading of Recommendations Assessment, Development and Evaluation; IS, information specialist; PPI, patient and public involvement.

### 3.2 Searches, screening, and extraction

#### 3.2.1 Search methods

LSR require explicit, predefined methods describing search frequency, and when and how new evidence will be incorporated. The evidence is continually under surveillance and new evidence should be flagged for the reader in a timely manner. This evidence should then be incorporated into the review at time points specified a priori or when the accumulated evidence is likely to change review conclusions.

We update searches for our LSR on the first working day of each month. We search MEDLINE, Embase, and PsycINFO via Ovid, using an automated search update function that returns every relevant record added since we last updated the search. This automation saves time running monthly search updates, minimizes the task of deduplication and helps to prevent delays, as updates are automatically shared with the account owner. We search CENTRAL using CRS Web, which also allows deduplication against records returned by previous searches. We save search results separately each month, and log how many results are returned by each database.

Monthly searches allow continual screening of evidence, and scrutiny of the search process, including the search strategy, in a rapidly evolving research field. Indexing terms and keywords may grow or change, and new search filters may be published.

#### 3.2.2 Screening of studies

Within 1 week of each monthly search, we screen citations generated using Covidence—a web-based systematic reviewing software—which allows automated deduplication of search results [22]. We carry out independent duplicate screening of titles, abstracts, and full texts. Allocation of screening to specific members of the author team and setting deadlines promotes timely completion. We name each search by month/year and keep each separate within Covidence. This aids monitoring and reporting, and allows monthly searches to continue once an update has been triggered. Where multiple papers are identified, reporting on the same study, these are grouped with one study ID. If additional citations are identified during later searches, these are classified as ‘new linked papers’. Each month papers identified are logged in a cumulative spreadsheet (recording: study ID; reference; study size; study design; comparator; names of extractors; notes; author e-mails), accessible to all of the core team, with new eligible citations classified as one of the following.

• A new included study.
• A new ongoing study.
• A new linked paper (classified further as ‘new data’ or ‘citation only’ based on whether additional data extraction is needed).

We track the progress of ongoing relevant studies, for example, through trial registries and contact with study investigators. When studies are at or nearing the specified study completion date, we contact investigators to ask whether findings are available.

#### 3.2.3 Data extraction

Data are extracted monthly for all new studies and any relevant linked papers. The process of data extraction is the same for LSRs as for systematic reviews. The extracted data aid our decisions on whether to update (see below) and allow timely publication when an update is triggered.

File management is important for the smooth running of the LSR process and when working with multiple authors. We use a prepiloted data extraction sheet. Two authors independently extract data, with discussion where there are disagreements, and agreed data extraction sheets are stored on a shared drive.

### 3.3 Review updates

#### 3.3.1 Deciding whether to trigger an update

Following screening each month, we assess whether the cumulative evidence found since the last published update would change the interpretation or certainty of our conclusions. There are three possible outcomes: (1) no new studies or information identified that would contribute to syntheses, (2) new studies or information identified but will have no relevant impact on conclusions, and (3) new records identified that would impact on conclusions [8]. Per-protocol, a full update of our review is carried out when new evidence changes one or more of the following review components: the interpretation or certainty (i.e., Grading of Recommendations Assessment, Development and Evaluation rating [4]) of one or more outcomes, or the range of settings, populations, interventions, comparisons, or outcomes investigated (Table 1). When unclear, we consult the wider author team to determine whether an update is justified.

#### 3.3.2 Monthly actions if an update is not triggered

Regardless of whether an update is triggered, we extract data and assess the risk of bias of any new studies. We share links to any new eligible studies: in online stakeholder briefings; in a spreadsheet on our webpage [23]; on social media; in our podcast series [24]; and, until March 2023, within the Cochrane Review under ‘version history’ [25].

#### 3.3.3 Actions if update is triggered

Where an update is triggered, we aim to have it published within 3 months of the date of the search that triggered it. This allows findings to be available to stakeholders in a timely fashion.

We conceptualize a full update as a train leaving the station. If in the months between the update being triggered and the publication of that update, new evidence is identified that would change conclusions again then the update or ‘train’ should be stopped to incorporate this new evidence. We developed clear prespecified ‘stopping rules’ that applied to our three main review comparisons (those most relevant to our stakeholders) and our critical outcomes only. The stopping rules are as follows: evidence identified will change the certainty or direction of effect of our smoking cessation outcome, evidence identified provides new signal of a serious adverse event attributable to EC, and evidence identified changes the direction of effect for the serious adverse event outcome so it indicates harm or leads to certainty being downgraded where the effect direction had indicated benefit.

#### 3.3.4 Updating review and editorial process

To speed up the manuscript reviewing process for co-authors and editorial staff, we suggest highlighting changes to the text that have occurred between updates. A short introduction and discussion with few specifics that require updating save time. The numbers of records screened, included, and excluded from each monthly search have to be combined to generate a flow diagram illustrating the process of incorporating the new evidence for each new update.

Authorship roles, conflicts of interest, permission forms, and funding statements need to be kept up-to-date so these can be submitted for editorial review quickly. These expectations, as well as those for timely review of the manuscript, should be made clear to the author team in advance to avoid delays. To co-ordinate the availability of the study team and editors, we alert them by e-mail as soon as an update is triggered. We provide a timeline of when they will need to read and edit the review and submit conflict of interest forms. One member of the team manages this process. We have an established relationship with the editorial base that is publishing the LSR, and an agreed process for the publication of updates.

#### 3.3.5 Dissemination activities

Our dissemination activities and materials are described fully in Table 2. These include stakeholder briefing documents, our podcast series [24], and our project webpage [23], all updated monthly. It also includes more long-term dissemination methods, such as short films, infographics, webinars, conference addresses, and academic publications. The podcast has more than 9,000 listeners in more than 53 countries since our first episode in December 2020. Our video summarizing our research and findings made with the University of Oxford Sparks team has received more than 38,000 views since going live in October 2022. We report survey feedback on our dissemination methods and materials in Supplementary Table 1. Being up-to-date with the evidence means we are prepared to respond to press enquiries and our research findings have been covered in more than 300 press reports worldwide, including the Economist, The New York Times, and the BBC. All materials and links to press coverage can be accessed from our webpage [23].

**Table 2:** Dissemination activities of the LSR for full review updates and monthly search findings

Dissemination activities for full updates	Dissemination activities monthly searches
• Update link to Cochrane review of EC for smoking cessation on ECLSR webpage (24). • Briefing documents: (1) Plain language briefing document. (2) Healthcare professional and policymakers briefing document. • Press releases. • Press interviews. • Short film(s) disseminating findings. • Infographic. • Webinar(s). • Plain language summary. • Conference and meeting addresses. • Impact updates on wider departmental website.	• Update ‘search update’ section of our two briefing documents to share new monthly search findings and the evidence accumulated since our last update. • Search updates on Cochrane Library in ‘version history’ section of review (until March 2023). • Spreadsheet of monthly search findings accessible from our webpage (from April 2023) (24). • Podcast series ‘Let us talk e-cigarettes’ with monthly search findings, summaries of relevant studies, and interviews with investigators of eligible studies. Oxford University podcast website (23) and available on iTunes and Spotify. • Regularly updated project webpage. • Monthly search findings shared on Twitter.
General dissemination materials that contain the main findings but do not include effect estimates or GRADE certainty ratings. Materials that will be used over the longer term, take time to create, or are more difficult to update, for example, • films • infographic • joint briefing with ASH (Action on Smoking and Health)	
Dissemination channels: social media, project webpage, and ASH newsletter.	

*Abbreviations*: LSR, living systematic review; EC, e-cigarette; ECLSR, e-cigarette living systematic review; GRADE, Grading of Recommendations Assessment, Development and Evaluation.

#### 3.3.6 Future of LSR

The need for a systematic review to be ‘living’ may change if findings become stable or the question is no longer deemed a priority for decision makers. For example, if the evidence was judged to be high certainty for all outcomes and all comparisons, consideration should be given to ceasing LSR status. If outcomes are not yet certain, discussions with the author team and stakeholders should be held to determine the next steps. Eighteen months into our LSR’s ‘living’ status, we evaluated the LSR approach, including the strengths and weaknesses of continuing this methodology for this evidence base, and asked stakeholders whether such an approach remained warranted. We carried out a patient and public involvement focus group and survey to ask for feedback on the future of the LSR (Supplementary Table 1). In May 2022, we consulted with our author team on the future of the LSR, considering survey and focus group findings. We decided to continue with the living format and seek further funding due to the continued policy need and uncertain outcomes, particularly related to EC safety.

## 4 Discussion

Historically, it was recommended that Cochrane systematic reviews be updated every 2 years, although realistically this generally occurred every 3–4 years. For EC, where the evidence base is rapidly evolving, this schedule impeded the ability of the review to provide the most up-to-date evidence to decision makers. Since converting our Cochrane Review of EC for smoking cessation to an LSR, our regular updates have facilitated strengthening of our conclusions and the amalgamation of evidence on new outcomes. We have implemented approved Cochrane LSR methods to provide an up-to-date, accessible, and unbiased review of the evidence [8]. The automated monthly search updates in Ovid save significant time. It also provides additional protection from unplanned absence disrupting the search update schedule. We have not encountered any difficulties using this standard feature in Ovid.

The LSR process means that the core review team is constantly up-to-date with the literature and evidence. This facilitates being reactive, making it easier to agree to be interviewed and talk regularly on the topic. When seeking to inform policy, it is important to be constantly ready to respond to requests for evidence-based advice.

The time commitment to conduct an LSR is high and must be seriously considered before embarking on, or transitioning to, an LSR. The approach is suited to fast-moving, policy-relevant topics where the evidence will feed into practice; where this is not the case, a standard systematic review may be more appropriate. COVID-19 research has been well suited to the LSR approach, indeed most LSRs relate to COVID-19 [2]. However, Cochrane LSRs also cover other topics, including treatment for chronic plaque psoriasis [26,27]. This Cochrane review of ‘Interventions for increasing fruit and vegetable consumption in children aged 5 years and under’ by Hodder et al. [28], transitioned to a living review in 2017 and was part of the LSR approach piloted by Cochrane. Since becoming a living review, four updates have been published. This review, Cochrane guidance, and an author with a background in LSR helped to shape the methodology for our LSR, including selecting monthly searches [[7], [8], [9], [10], [11], [12], [13], [14], [15], [16], [17], [18],^28^]. Other LSRs differ in their approaches and screening is carried out at different time intervals, for example, the LSR by Zheng et al. [2] on convalescent plasma for people with COVID-19 carried out weekly searches until August 2021 and have now transitioned to monthly searches. A review by Spurling et al. [29] on delayed antibiotic prescriptions for respiratory infections transitioned to an LSR with monthly searches from May 2017, and then in August 2022, the authors decided to cease maintaining their review in LSR mode as a reasonable level of certainty had been reached in the existing evidence. We have learned from other reviews and hope that this paper and the tips in Table 1 section 4 will be helpful to others.

This paper is based on our experiences with one review and that experience might be specific to topic area, nature of the team, and availability of resources/funding. We are not able to report on retirement from living mode as we have not experienced this, reflecting the continued uncertainty and ongoing interest in this topic. There is an opportunity for further research evaluating the impact of the living process on the use of our review.

## 5 Conclusion

LSRs are relevant when the research question is of high importance for policy makers, there is uncertainty in the evidence base, and new evidence is likely to emerge that will change the certainty of evidence. If taken step-by-step and planned and implemented in a well-organized manner, an LSR process, with multiple timely updates, is achievable.

**Key messages**

**What is already known on this subject?**
• Living systematic reviews (LSRs) are systematic reviews that are regularly updated, allowing for new evidence to be incorporated as it becomes available.
• LSRs were proposed in 2014, as a method to regularly update reviews where the evidence remains uncertain, the topic is fast moving, and changes in the interpretation and certainty of the evidence may affect policy and healthcare decisions.
• More than half of published LSRs do not publish an update (1).

**What this study adds?**
• This paper highlights the need for an organized preplanned approach to the living systematic review (LSR) process. This is a time intensive, detail orientated endeavor and so should be embarked upon with consideration of a range of important factors, such as justification for the ‘living’ approach, the regularity of search updates, specification of triggers for full review updates and the termination of a living approach, and the methods through which stakeholders will be regularly informed of the emerging evidence.

**How this study might affect research, practice, or policy?**
• We aim to make the process of carrying out an LSR transparent by sharing our experience of both the review process and the dissemination of our findings to assist other researchers planning to carry out LSR and increase the proportion of LSR publishing more than once. We hope this will help researchers to carry out updates in a timely fashion, thereby enabling stakeholders to have access to the most up-to-date evidence for decision-making.

## CRediT authorship contribution statement

**Ailsa R. Butler:** Conceptualization, Data curation, Methodology, Project administration, Writing – original draft, Writing – review & editing, Visualization. **Jamie Hartmann-Boyce:** Conceptualization, Funding acquisition, Methodology, Writing – review & editing, Data curation, Writing – original draft, Supervision. **Jonathan Livingstone-Banks:** Conceptualization, Data curation, Funding acquisition, Methodology, Software, Writing – review & editing. **Tari Turner:** Conceptualization, Data curation, Methodology, Writing – review & editing. **Nicola Lindson:** Conceptualization, Data curation, Funding acquisition, Methodology, Supervision, Writing – original draft, Writing – review & editing.

## Declaration of competing interest

A.R.B., J.H.B., J.L.B., T.T., and N.L. have no conflicts of interest to declare.

## Data Availability

No data were used for the research described in the article.

## References

[1] Elliott J.H., Synnot A., Turner T., Simmonds M., Akl E.A., McDonald S. (2017). Living systematic review: 1. Introduction-the why, what, when, and how. J Clin Epidemiol.

[2] Zheng Q., Xu J., Gao Y., Liu M., Cheng L., Xiong L. (2022). Past, present and future of living systematic review: a bibliometrics analysis. BMJ Glob Health.

[3] World Health Organisation (2021).

[4] Schünemann H.J., Brożek J., Guyatt G., Oxman A. (2013). https://gdt.gradepro.org/app/handbook/handbook.html.

[5] Hartmann-Boyce J., McRobbie H., Bullen C., Begh R., Stead L.F., Hajek P. (2016). Electronic cigarettes for smoking cessation. Cochrane Database Syst Rev.

[6] Chen Z., Luo J., Li S., Xu P., Zeng L., Yu Q. (2022). Characteristics of living systematic review for COVID-19. Clin Epidemiol.

[7] EQUATOR. EQUATOR (2023). https://www.equator-network.org/reporting-guidelines-study-design/systematic-reviews-and-meta-analyses/?post_type=eq_guidelines.

[8] (2019). Guidance for the production and publication of Cochrane living systematic reviews: Cochrane Reviews in living mode. [Internet].

[9] (2023). Cochrane. Production resources: Living systematic reviews: Cochrane.

[10] Simmonds M., Elliott J.H., Synnot A., Turner T. (2022). Living systematic reviews. Methods Mol Biol.

[11] Turner T., McDonald S., Wiles L., English C., Hill K. (2022). How frequently should "living" guidelines be updated? Insights from the Australian Living Stroke Guidelines. Health Res Pol Syst.

[12] Cheyne S., Fraile Navarro D., Buttery A.K., Chakraborty S., Crane O., Hill K. (2023). Methods for living guidelines: early guidance based on practical experience. Paper 3: selecting and prioritizing questions for living guidelines. J Clin Epidemiol.

[13] Cheyne S., Fraile Navarro D., Hill K., McDonald S., Tunnicliffe D., White H. (2023). Methods for living guidelines: early guidance based on practical experience. J Clin Epidemiol.

[14] Fraile Navarro D., Cheyne S., Hill K., McFarlane E., Morgan R.L., Murad M.H. (2023). Methods for living guidelines: early guidance based on practical experience. Article 5: decisions on methods for evidence synthesis and recommendation development for living guidelines. J Clin Epidemiol.

[15] Millard T., Synnot A., Elliott J., Green S., McDonald S., Turner T. (2019). Feasibility and acceptability of living systematic reviews: results from a mixed-methods evaluation. Syst Rev.

[16] Bendersky J., Auladell-Rispau A., Urrútia G., Rojas-Reyes M.X. (2022). Methods for developing and reporting living evidence synthesis. J Clin Epidemiol.

[17] Metzendorf M.I., Weibel S., Reis S., McDonald S. (2022). Pragmatic and open science-based solution to a current problem in the reporting of living systematic reviews. BMJ Evid Based Med.

[18] Kahale L.A., Elkhoury R., El Mikati I., Pardo-Hernandez H., Khamis A.M., Schünemann H.J. (2021). Tailored PRISMA 2020 flow diagrams for living systematic reviews: a methodological survey and a proposal. F1000Res.

[19] Lindson N., Richards-Doran D., Heath L., Hartmann-Boyce J. (2017). Setting research priorities in tobacco control: a stakeholder engagement project. Addiction.

[20] James Lind Alliance (2021). https://www.jla.nihr.ac.uk/jla-guidebook/downloads/JLA-Guidebook-Version-10-March-2021.pdf.

[21] Hartmann-Boyce J., McRobbie H., Lindson N., Bullen C., Begh R., Theodoulou A. (2020). Electronic cigarettes for smoking cessation. Cochrane Database Syst Rev.

[22] Covidence Covidence systematic review software, Veritas Health Innovation, Melbourne, Australia. https://www.covidence.org.

[23] Electronic cigarettes for smoking cessation: cochrane living systematic review webpage. https://www.cebm.ox.ac.uk/research/electronic-cigarettes-for-smoking-cessation-cochrane-living-systematic-review-1.

[24] Let’s talk e-cigarettes podcast series. https://podcasts.ox.ac.uk/series/lets-talk-e-cigarettes.

[25] Hartmann-Boyce J., Lindson N., Butler A.R., McRobbie H., Bullen C., Begh R. (2022). Electronic cigarettes for smoking cessation. Cochrane Database Syst Rev.

[26] Iannizzi C., Chai K.L., Piechotta V., Valk S.J., Kimber C., Monsef I. (2023). Convalescent plasma for people with COVID-19: a living systematic review. Cochrane Database Syst Rev.

[27] Sbidian E., Chaimani A., Guelimi R., Garcia-Doval I., Hua C., Hughes C. (2023). Systemic pharmacological treatments for chronic plaque psoriasis: a network meta-analysis. Cochrane Database Syst Rev.

[28] Hodder R.K., O'Brien K.M., Tzelepis F., Wyse R.J., Wolfenden L. (2020). Interventions for increasing fruit and vegetable consumption in children aged five years and under. Cochrane Database Syst Rev.

[29] Spurling G.K.P., Del Mar C.B., Dooley L., Clark J., Askew D.A. (2017). Delayed antibiotic prescriptions for respiratory infections. Cochrane Database Syst Rev.

