# Supplementary material for: Optimizing process and methods for a living systematic review: 30 search updates and three review updates later
Source: J Clin Epidemiol. 2024 Feb;166:None. doi: 10.1016/j.jclinepi.2023.111231 (PMC12018299; doi:10.1016/j.jclinepi.2023.111231)
Supplement: Supplementary Table 1 [file mmc1.docx]

Supplementary Table 1: Survey to gather feedback on our review, supporting materials, dissemination methods and future of the LSR. Survey questions and summary of responses

|  | Question | Summary of responses |
| --- | --- | --- |
| 1 | Are you aware of the Cochrane review of electronic cigarettes for smoking cessation? | 92% of respondents reported that they were aware of the review. |
| 2 | At the moment we search the literature every month. Do you think we should continue to search regularly for information? | 98%, of respondents considered that we should continue to search regularly for information. |
| 3 | If you think we should continue to search for information how often should we do this? | 66% of respondent answered every month, 17% every 2 months, 14% every 3 months, 5% every 6 months and 1% every year. No one thought we should search every 2 years or more. |
| 4 | How useful you find the following materials on a scale of 1 to 5? Where 1 is not useful and 5 is useful   1. Cochrane review of electronic cigarettes for smoking cessation 2. Briefing documents 3. Podcast 4. Webpage | 96% of respondents gave the Cochrane Review a ranking of 4 or above. Only 3% of respondents said they did not find this publication useful.  91% gave the briefing documents a ranking of 4 or above. 2% did not find the briefing documents useful.  65% gave the podcast a ranking of 4 or above. 5% did not find the podcast useful.  87% gave the webpage a ranking of 4 or above. 3% did not find the webpage useful. |
| 5 | Do you have examples of where you have used the Cochrane review of e-cigarette for smoking cessation or the supporting documentation? | 44% of respondents provided examples of where they had used the Cochrane review or supporting materials. Respondents used this to inform people interested in quitting, government policy, the public, health care providers, clinical colleagues, and media reports. |
| 6 | If you listen to the podcast is there anything that you would like us to do differently? | 44 respondents provided feedback. 21/44 thought these should continue as they were/enjoyed the podcast. |
| 7 | Can you think of anything else we should be doing or anything we should be doing differently? | 47 people responded to this question. 16 respondents said to continue as we are doing.  Other feedback included suggestions for additional ways of disseminating review findings, specific to this topic. |

197 responses received from people who used combustible cigarettes or vapes, people providing stop smoking support, researchers, members of relevant charities or advocacy groups and policy makers.

Survey was piloted with members of the public and colleagues in the research team. All questions were optional and had a free text box.

Survey tool JISC Online Survey.

Podcasts series link: <https://podcasts.ox.ac.uk/series/lets-talk-e-cigarettes>
